# Comparative analysis of fruit aroma patterns in the domesticated wild strawberries “Profumata di Tortona” (*F. moschata*) and “Regina delle Valli” (*F. vesca*)

**DOI:** 10.3389/fpls.2015.00056

**Published:** 2015-02-11

**Authors:** Alfredo S. Negri, Domenico Allegra, Laura Simoni, Fabio Rusconi, Chiara Tonelli, Luca Espen, Massimo Galbiati

**Affiliations:** ^1^Dipartimento di Scienze Agrarie e Ambientali - Produzione, Territorio, Agroenergia, Università degli Studi di MilanoMilan, Italy; ^2^Plant Model System Platform, Fondazione FilareteMilan, Italy; ^3^Department of Life Sciences, Università degli Studi di MilanoMilan, Italy

**Keywords:** strawberry, *Fragaria moschata*, *Fragaria vesca*, gas chromatography-mass spectrometry, aroma, volatiles

## Abstract

Strawberry is one of the most valued fruit worldwide. Modern cultivated varieties (*Fragaria* × ananassa) exhibit large fruits, with intense color and prolonged shell life. Yet, these valuable traits were attained at the cost of the intensity and the variety of the aroma of the berry, two characteristics highly appreciated by consumers. Wild species display smaller fruits and reduced yield compared with cultivated varieties but they accumulate broader and augmented blends of volatile compounds. Because of the large diversity and strength of aromas occurring in natural and domesticated populations, plant breeders regard wild strawberries as important donors of novel scented molecules. Here we report a comprehensive metabolic map of the aroma of the wild strawberry Profumata di Tortona (PdT), an ancient clone of *F. moschata*, considered as one of the most fragrant strawberry types of all. Comparison with the more renowned woodland strawberry Regina delle Valli (RdV), an aromatic cultivar of *F. vesca*, revealed a significant enrichment in the total level of esters, alcohols and furanones and a reduction in the content of ketones in in the aroma of PdT berries. Among esters, particularly relevant was the enhanced accumulation of methyl anthranilate, responsible for the intensive sweetish impression of wild strawberries. Interestingly, increased ester accumulation in PdT fruits correlated with enhanced expression of the *Strawberry Alcohol Acyltransferase* (*SAAT*) gene, a key regulator of flavor biogenesis in ripening berries. We also detected a remarkable 900-fold increase in the level of mesifurane, the furanone conferring the typical caramel notes to most wild species.

## Introduction

Garden strawberries (*Fragaria* × ananassa) are among the most appreciated fruits and represent a valuable economic crop with a global annual production that exceeds 4.5 Mt (FAOSTAT, 2014). Berries of high yield modern varieties are characterized by large size, attractive color and prolonged shell life (Hancock, 1999). Yet, the sensory quality of traded strawberries is often criticized, as they lack flavor and fragrance. Sensory perceptions originate from the combination of sweetness, texture and aroma (Christensen, 1983). Among these features, aroma remains the most valued quality indicator for consumers worldwide (Azodanlou et al., 2003; Colquhon et al., 2012). Just as in other fruits, strawberry's aroma is a complex blend of volatile organic compounds (VOCs). These compounds only represent 0.001 to 0.01% of the berry fresh weight but have a major effect on its flavor and fragrance (Buttery, 1983). As many as 360 volatiles have been identified in ripe strawberries; these include esters, aldehydes, ketones, alcohols, terpenes and furanones (Menager et al., 2004; Jetti et al., 2007). Individual compounds, although often present in minute quantities, may have a significant impact on the aroma. The reduced fragrance of most garden strawberries derives from the relatively limited accumulation of esters molecules, frequently combined with an excess of lactones, which usually cause a disproportionate peach note.

As opposite to garden varieties, wild strawberries are renowned for their intense flavor and fragrance. Most spontaneous species bear small fruits which accumulate higher levels and wider assortments of volatile molecules, compared with cultivated varieties (Honkanen and Hirvi, 1990). The ample natural variation occurring among the wild ancestors of garden strawberries provides a valuable source of novel volatile compounds for breeding new commercial strawberries with improved aroma properties (Ulrich and Hoberg, 2000a). Over 20 wild species are found within the *Fragaria* genus, of which the diploid woodland strawberry (*F. vesca*) is the most common (Rousseau-Gueutin et al., 2009). Among other species, musk strawberries (*F. moschata*) are recognized for their distinguished and extraordinary strong aroma. Native to highland areas from France to Siberia, musk strawberries were widely cultivated in Europe to the mid-1900, when they were replaced by firmer, higher yielding and more remunerative *F*. × ananassa cultivars (Darrow, 1966).

Today, only few musk strawberries survive in farm plantings, although on a very small scale. Noteworthy is the Italian clone Profumata di Tortona (PdT), regarded as one of the most fragrant strawberry types of all (Urruty et al., 2002). PdT is a dioecious strawberry having the male and female reproductive organs in separate flowers on separate plants. Berries, distinguishable for the intense red color of the peel and the whitish flesh, posses a delightful sour–sweet, slightly astringent flavor, with green, caramel and clove-like notes (Pet'ka et al., 2012). Hallmark of PdT is its peculiar floral, spicy aroma, with hints of honey, musk and wine. The fragrance of this strawberry is so intense that a few ripe berries can perfume an entire room with a penetrating mango-like, tropical scent. Differently from most cultivated strawberries, the harvesting season for PdT is extremely limited. Berries are only available for a period of 10–15 days, coinciding with the second half of June. Currently, the commercial cultivation of PdT is restricted to the municipality of Tortona, in the Pedimont region in Northern Italy. Remarkably, the first historical evidence of musk strawberries in this area dates back to year 1411 (Bergaglio, 2007). Cultivation lingered into the early 1960s, when strawberry fields succumbed to urban development. Most recently, there has been a renewed interest for the Profumata di Tortona, considered a delicacy both for fresh consumption and gourmet preparations.

Diversity of volatile patterns in woodland strawberries in comparison to cultivated garden varieties has been extensively investigated (Drawert et al., 1973; Ulrich et al., 1997). Recent surveys of aroma profiles across 16 *F. vesca* accessions and five *F*. × ananassa cultivars, identified significant differences in the accumulation of individual esters, ketones and terpinoids between the two strawberries (Ulrich and Olbricht, 2013, 2014). In particular, small esters, including ethyl hexanoate, methyl butanoate and methyl hexanoate, were found in higher amounts in garden strawberries compared with woodland accessions. Conversely, the key ester methyl antranilate (MA) was more abundant in *F. vesca*. Similarly, ketones (e.g., 2-pentanone, 2-heptanone, and 2-nonanone) and terpinoids (e.g., myrtenal, myrtenil acetate, α-terpineol) occurred at higher levels in wild berries, with the exception of the monoterpene linalool, which was more abundant in garden strawberries (Ulrich and Olbricht, 2013, 2014).

Detailed profiling of the aroma composition in musk strawberries has only been reported for few spontaneous populations (Ulrich et al., 2007; Pet'ka et al., 2012). Urruty and colleagues performed a first partial assessment of the volatile compounds produced by ripe PdT berries (Urruty et al., 2002). These authors determined the abundance of 23 preselected VOCs in the aroma of two *F. moschata* clones (Capron Royal and PdT) compared with 15 garden varieties. Selected compounds, representing major constituents of the strawberry aroma, included esters (e.g. methyl hexanoate, MA), monoterpenes (e.g., linalool, nerolidol), ketones (e.g., 2-pentanone, 2-heptanone), aldehydes (e.g., 2-hexenal), lactones and furanones (e.g., γ-decalactone, mesifurane). Among the volatiles analyzed, Capron Royal and PdT displayed lower levels of small esters as methyl hexanoate, compared with cultivated strawberries. In contrast, both *F. moschata* clones revealed exceptionally high levels of MA, which was barely detectable in most garden varieties (Urruty et al., 2002). Despite the relevance of these findings, a more comprehensive analysis of the aroma profile of PdT is required to fully uncover the volatile composition of ripe PdT berries and gain more insights into its extraordinary aromatic properties.

Here we report a re-assessment of the VOCs composition of PdT berries, based on a non-targeted Solid-Phase Micro-Extraction/Gas chromatography-Mass Spectrophotometry (SPME/GC-MS) approach (Ulrich and Hoberg, 2000b). We compared the aroma of PdT with that of the woodland strawberry Regina delle Valli (RdV). The latter represents a widely cultivated cultivar of *F. vesca*, renowned for its intense and pleasant aroma. Most importantly, the aroma composition of this strawberry has not been investigated in previous studies. In total, we identified 131 VOCs in the headspace of the two strawberries, which provide a comprehensive picture of the aroma patterns of PdT and RdV berries. As a whole, our results contribute to shed new light on the natural variation occurring in the aroma of wild strawberry species.

## Materials and methods

### Plant material

Plants of Profumata di Tortona were provided by “Consorzio per la valorizzazione e la tutela della Fragola Profumata di Tortona,” Tortona, Italy. Regina delle Valli plants were purchased from Azienda Agricola Ortomio, Forlì, Italy (http://www.ortomio.it/). Both strawberries were grown in the production area of Tortona (Italy) under commercial conditions, accordingly to the standards adopted by the “Consorzio per la valorizzazione e la tutela della Fragola Profumata di Tortona.” Fruits for the analysis were harvested with the assistance of local producers, to ensure selection of uniform, healthy and fully ripe berries. Five randomized samples, composed of 15 individual ripe fruits each, were collected the early morning in a single harvest. The extremely reduced harvesting season of PdT did not justify the adoption of multiple harvests. Berries employed in our analysis represent a faithful sample of the commercial fruits that are normally available to consumers.

### SPME/GC-MS sample preparation

Harvested fruits samples were immediately frozen at −20°C and stored at −80°C. Prior to the analysis fruits were powdered in liquid nitrogen and 1 g of fresh weight for each sample was incubated at 30°C for 5 min. Following addition of 300 μ L of a NaCl saturated solution, 900 μ L of the homogenized mixture were transferred to a10 mL screw cap headspace vial. Three technical replicas were performed for each sample.

### Automated SPME/GC-MS

Volatiles were sampled by SPME with a 2 cm × 50/30-micron DVB/Carboxen/PDMS Stable Flex fiber (Sigma, Milano, Italy). Extraction and desorption of the volatiles were performed automatically by a CombiPAL autosampler (CTC Analytics, Zwingen, Switzerland) as described (Zorrilla-Fontanesi et al., 2012). Chromatography was performed on a DB-5 ms (30 m × 0.25 mm × 1 mm) column (Sigma, Milano, Italy) with Helium at a constant flow of 1.2 mL/min, accordingly to Zorrilla-Fontanesi et al. (2012). Mass spectra were recorded in scan mode in the 35 to 220 mass-to-charge ratio range by a 5975B mass spectrometer (Agilent Technologies, Cernusco sul Naviglio, Italy) (ionization energy 70 eV; scanning speed 7 scans/s). The Enhanced ChemStation software (Agilent Technologies, Cernusco sul Naviglio, Italy) was used for recording and processing of chromatograms and spectra. Three technical replicas were conducted for each sample.

### Compound identification and relative quantification

Compound abundance was determined using the software MET-IDEA that directly extracts ion intensities exploiting a list of ions coupled with their relative retention time values (Broeckling et al., 2006). The ion list was built as following. For each biological replicate, a randomly chosen chromatogram was analyzed by Automated Mass Spectral Deconvolution and Identification System (AMDIS; http://chemdata.nist.gov/dokuwiki/doku.php?id=chemdata:amdis) comparing the deconvoluted spectra with entries at the National Institute of Standards and Technology MS library (NIST08) (Type of Analysis, Simple; Resolution, Medium; Sensitivity, Medium; Shape Requirement, Low; Component Width, 12; Minimum Match Factor, 70). The 113 entries with a match score larger than 800 on the NIST Search software were extracted and added to a.msl file containing the 1537 plant derived metabolites of the VOC BinBase library (Skogerson et al., 2011) to assemble the library used for the analysis of the 10 randomly-chosen chromatograms. During this second cycle of AMDIS analysis, the Minimum match Factor was set to 90 and the resulting.fin files were joined in a single ion list, manually eliminating overlapping entries. The resulting ion list was employed for Met-IDEA analysis over all the 30 chromatograms. Peak areas of selected specific ions were integrated for each compound. The relative content (R.C.) of each tentatively identified metabolite (expressed as percentage) was calculated as the ratio between each peak area and the sum of all the peak areas present in the chromatogram, multiplied by 100 [R.C. = (Area_peak_/ΣAreas_peak_) × 100]. CAS numbers and flavoring descriptors were retrieved from the web-based Chemical Search Engine (http://www.chemindustry.com/apps/chemicals) and from the online edition of the “Specifications for Flavorings” database (http://www.fao.org/ag/agn/jecfa-flav/), respectively.

### Statistical analysis

Differences in volatiles accumulation between the two cultivars were investigated through the Soft Independent Modeling of Class Analogies (SIMCA) provided by the software Unscrambler (Camo Process AS, Oslo, Norway), trough the construction of a Principal Component Analysis (PCA) model for each cultivar. Samples were projected on the orthogonal system constituted by the two models to assess their object-to-model distance and to judge their membership to one of the two classes. The capability of variable *k* in discriminating between model PdT and RdV (fitting samples from model PdT onto model RdV) was described by the Discrimination Power (DiscrPower), computed as:

$$\text{DiscrPower}=\frac{{\text{S}}_{\text{PdT}}{\left(\text{RdV},\text{ k}\right)}^{2}+{\text{S}}_{\text{RdV}}{\left(\text{RdV},\text{ k}\right)}^{2}}{{\text{S}}_{\text{RdV}}{\left(\text{RdV},\text{ k}\right)}^{2}+{\text{S}}_{\text{PdT}}{\left(\text{PdT},\text{ k}\right)}^{2}}$$

The significance of the differences in volatile levels in the two strawberries was tested through a *t*-Student test considering as significant variations with *p* < 0.01.

### Quantitative PCR (qPCR) analysis

RNA was isolated from Small Green, Turning and Red fruits according to Schultz et al. (1994). Reverse transcription, and qPCR analysis were performed as previously described (Galbiati et al., 2011). *SAAT* expression was analyzed using primers SAAT-F1 (5′-TTGGATGGGGGAGGACATCAT-3′) and SAAT -R1 (5′-CACCCACGCTTCAATTCCAGTA-3′). Gene expression was normalized using the *ACTIN* gene (GenBank: JN616288.1), amplified with primers ACT-F1 (5′-ATGTTGCCCTTGACTACGAACAA-3′) and ACT-R1 (5′- TGGCCGTCGGGAAGCTCATA-3′). Primers efficiency was first assessed on both genomic DNA and cDNAs derived from PdT and RdV fruits, to avoid differences in amplification efficiency in the two genotypes. Changes in *SAAT* gene expression were calculated relative to *ACTIN* using the ΔΔCt method (Livak and Schmittgen, 2001).

## Results

### PdT and RdV berries display distinct VOCs patterns

**Figure 1:**
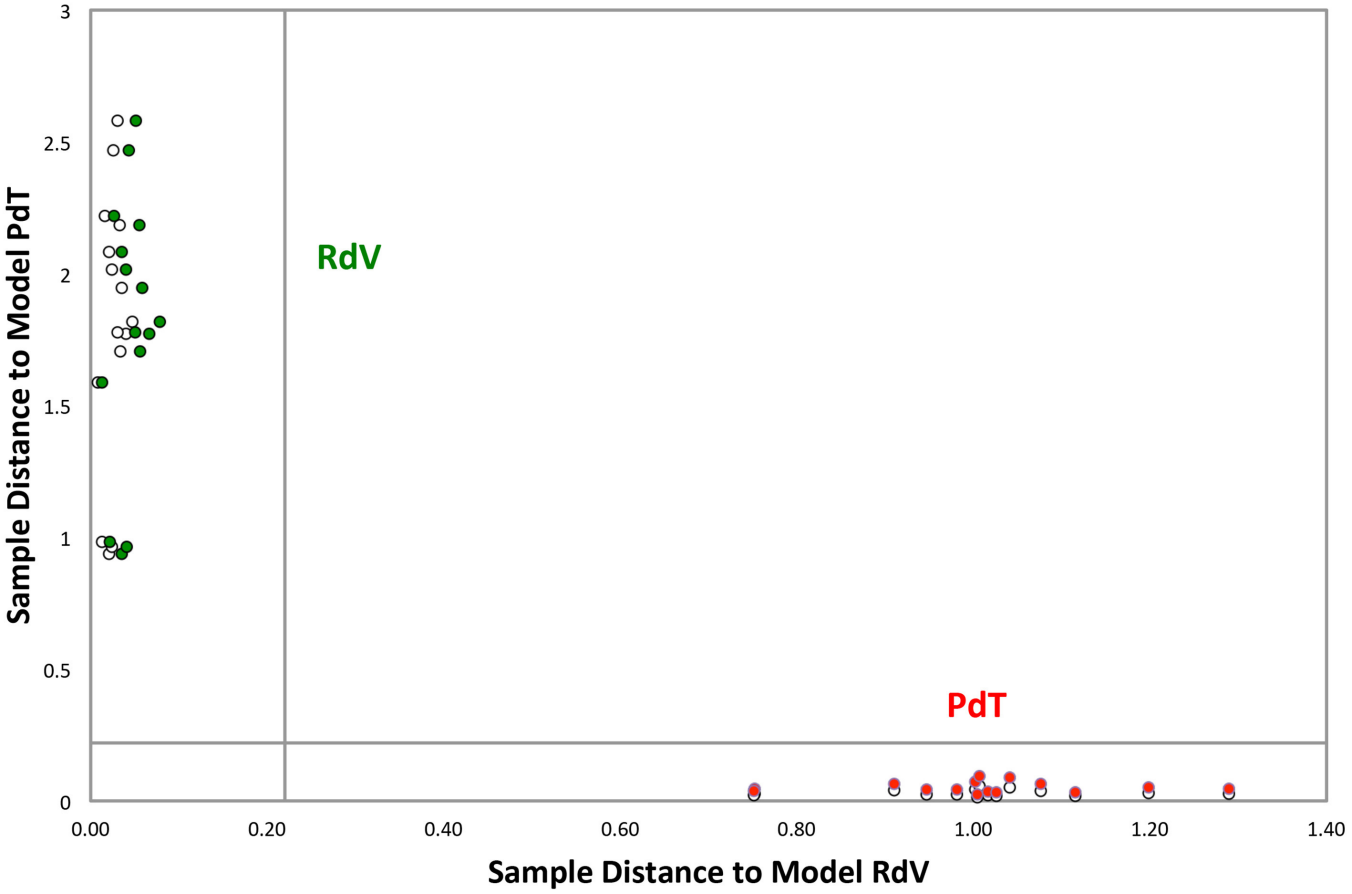
**Cooman's plot of the classification analysis between PdT and RdV**. Green and red circles represent calibration samples belonging to RdV and PdT, respectively. White circles represent test set samples of the two classes. Graph inner lines represent the significance level of 5%. The analysis employed 131 peak signals. Among these, 95 peaks were tentatively identified by MS library search.

Fully ripe berries of *F. moschata*, clone Profumata di Tortona (PdT) and *F. vesca*, cv Regina delle Valli (RdV) were analyzed by SPME/GC-MS. In total, through the construction of a non-redundant ion list collecting information from the AMDIS analysis of 10 different chromatograms, 131 VOCs were tentatively identified in the headspace of the two strawberries. GC-MS data obtained from individual biological and technical replicas were analyzed using the Soft Independent Modeling of Class Analogies (SIMCA) based on the models relative to the two genotypes built with the Principal Component Analysis (PCA) (Svante and Michael, 1977). The SIMCA Cooman's plot showed full differentiation of PdT and RdV, based on their aroma components. All the samples grouped in the relative membership class, as determined by a significance level of 5% (Figure 1). The model assigned the highest discriminating power to the ketones heptan-2-one and nonan-2-one (Table 1). A third ketone molecule, undecan-2-one, also scored among the most significant volatiles for the discrimination between the two strawberries. Additional molecules with high discriminating power included several esters, such as hexyl butanoate, methyl benzoate, ethyl hexanoate, pinocarvyl acetate, the furanone γ-hexalactone, and the terpenes pinocarveol and myrtenyl acetate (Table 1).

**Table 1:** **Volatiles with the highest discriminating power identified by the SIMCA model**.

**Compound**	**Discriminating power**	**Compound**	**Discriminating power**
heptan-2-one	317.183	[(Z)-hex-3-enyl] acetate	61.793
nonan-2-one	201.421	(1Z,4Z,7Z)-1,5,9,9-tetramethylcycloundeca-1,4,7-triene	60.002
hexyl butanoate	106.238	alpha-Ionyl acetate	55.384
methyl benzoate	87.871	4-(2,6,6-trimethylcyclohex-2-en-1-yl)butan-2-yl acetate	55.077
2-methylbutanoic acid	81.037	methyl 2-methylbutanoate	54.337
ethyl hexanoate	75.867	methyl decanoate	54.171
pinocarvyl acetate	75.321	decyl acetate	51.343
octyl acetate	73.294	4-acetyloxybutyl acetate	51.341
undecan-2-one	69.701	pinocarveol	49.644
γ-Hexalactone	65.968	myrtenyl acetate	48.208
butyl butanoate	65.954	4-acetyloxypentan-2-yl acetate	48.201
methyl tiglate	65.668	hexyl acetate	47.006

### Comparative analysis of aroma patterns in PdT and RdV berries

Among the 131 volatiles identified in the aroma of the two strawberries, 47 were classified as esters, which are long known to represent the most abundant VOCs found in ripe strawberries (Latrasse, 1991) (Table 2). Terpenoids included 20 mono- and 4 sesqui-terpenes (Table 3). Amongst the remaining aroma constituents we identified 5 alcohols, 9 aldehydes (Table 4), 6 ketones, and 4 lactones (Table 5). Additional compounds comprised a single fatty acid (hexanoic acid), a single alkane hydrocarbon (tetradecane), and 36 volatiles of unknown chemical identity.

**Table 2:** **Ester molecules identified in the headspace of PdT and RdV berries by GC-MS**.

**Ester compound**		**Quantification Ion**	**Retention time**	**CAS**	**Descriptor**	**Relative Content (%)**		**Fold change**	***p*-value**
**IUPAC name**	**Synonym**					**PdT**	**RdV**		
octyl acetate		43.1	19.8167	112-14-1	Fruity, orange-like, jasmine-like odor	12.687	4.644	2.73	2.152E-07
4-acetyloxybutyl acetate		43.1	19.878	628-67-1		11.564	3.645	3.17	1.374E-06
hexyl formate		56.1	9.3341	629-33-4	Ethereal, fruity, leafy, green odor	4.555	1.292	3.53	1.017E-04
methyl 2-aminobenzoate	methyl anthranilate	119	23.4659	134-20-3	Grape-like or orange aroma	3.395	0.373	9.10	1.983E-05
hexyl acetate		43.1	13.9064	88230-35-7	Fruity odor	2.717	3.766	0.72	7.554E-02
2-methylbutanoic acid		74	9.76	600-07-7		1.805	0.015	120.33	1.221E-03
(7,7-dimethyl-4-methylidene-3-bicyclo[3.1.1]heptanyl) acetate	pinocarvyl acetate	92.1	17.8485	33045-02-2		1.348	0.591	2.28	6.287E-08
[(Z)-hex-2-enyl] acetate		67.1	13.9831	56922-75-9		1.087	1.018	1.07	3.949E-01
[(Z)-hex-3-enyl] acetate		67.1	13.7052	3681-71-8	Powerful green note	0.968	0.414	2.34	1.535E-03
(E)-hex-2-enyl] butanoate		43.1	19.3505	53398-83-7	Fruity, green aroma	0.752	0.784	0.96	4.126E-01
methyl 2-methylbutanoate		85.1	6.5488	868-57-5	Sweet, fruity, apple-like odor	0.751	0.005	150.20	1.577E-08
phenylmethyl acetate		108	18.485	140-11-4	Sweet, fruity, floral (Jasmine) odor	0.677	2.765	0.24	1.914E-04
methyl octanoate		74	17.3418	67762-39-4	Winey, fruity, orange odor	0.549	0.330	1.66	4.148E-02
decyl acetate		55.1	25.0756	112-17-4	Pear, floral, orange-rose odor	0.337	0.472	0.71	4.107E-03
tridecan-2-yl acetate		55.1	30.2373	not available		0.300	0.022	13.64	2.966E-09
heptan-2-yl acetate		43.1	14.8247	5921-82-4		0.299	0.238	1.26	2.395E-01
methyl benzoate		105	16.538	93-58-3	Pungent, heavy, floral odor with fruity undertones	0.247	0.174	1.42	7.030E-02
octyl 3-methylbutanoate		103.1	25.7912	7786-58-5	Apple-pineapple odor	0.235	0.015	15.67	8.846E-07
methyl (E)-2-methylbut-2-enoate	methyl tiglate	114	9.2297	6622-76-0		0.215	0.017	12.65	4.559E-07
[(E)-hex-2-enyl] propanoate		57.1	16.8525	53398-80-4	Fruity, ripe apple-pear aroma	0.159	0.073	2.18	4.585E-03
(2,4-ditert-butylphenyl) 5-hydroxypentanoate		191.1	27.6179	not available		0.135	0.218	0.62	4.427E-02
3-hydroxy-2,2,4-trimethylpentyl) 2-methylpropanoate		89	24.2546	80525-37-7		0.123	0.167	0.74	8.474E-02
hexyl butanoate		89.1	19.2782	2639-63-6	Fruity, apricot odor	0.111	0.444	0.25	1.173E-03
butyl butanoate		71	13.3589	109-21-7	Fruity, pineapple-like odor	0.089	0.853	0.10	3.059E-04
methyl decanoate		74	22.9111	110-42-9		0.073	0.181	0.40	3.490E-04
[2,2,4-trimethyl-1-(2-methylpropanoyloxy) pentan-3-yl] 2-methylpropanoate		71.1	29.6078	6846-50-0		0.072	0.085	0.85	2.162E-01
2-phenylethyl acetate		104	21.1851	103-45-7	Very sweet, rosy-fruity honey-like odor	0.071	0.037	1.92	1.962E-05
methyl dodecanoate		74	27.8978	111-82-0	Fatty, floral, winey odor	0.054	0.008	6.75	5.111E-04
methyl (E)-oct-2-enoate		55.1	18.6982	7367-81-9	Fruity, green aroma	0.052	0.019	2.74	7.342E-04
(Z)-hex-3-enyl] butanoate		67.1	19.1306	16491-36-4	Green, fruity, buttery odor	0.048	0.032	1.50	8.847E-03
[(E)-3-phenylprop-2-enyl] acetate		161	22.5682	103-54-8	sweet, balsamic, floral odor	0.048	0.002	24.00	1.191E-06
ethyl hexanoate		117.1	24.4915	8068-81-3	Wine-like odor	0.047	0.953	0.05	4.466E-02
(1-hydroxy-2,4,4-trimethylpentan-3-yl) 2-methylpropanoate		71.1	23.7336	74367-33-2		0.045	0.075	0.60	4.326E-02
octyl hexanoate		99.1	29.2171	4887-30-3		0.038	0.016	2.38	1.072E-04
hexyl hexanoate		117.1	24.4915	6378-65-0	Herbaceous odor	0.034	0.029	1.17	1.677E-01
4-(2,6,6-trimethylcyclohex-2-en-1-yl)but-3-en-2-yl acetate	alpha-Ionyl acetate	180.1	27.2825	94224-42-7		0.030	0.007	4.29	1.685E-06
ethyl octanoate		88	19.4368	106-32-1	Wine, brandy, fruity floral odor	0.029	0.776	0.04	4.239E-02
4-acetyloxypentan-2-yl acetate		103	18.1022	7371-86-0		0.029	0.003	9.67	1.321E-06
(4-propan-2-ylphenyl)methyl acetate		107	25.4881	59230-57-8		0.027	0.007	3.86	1.471E-15
ethyl decanoate		88	24.7276	110-38-3	Oily brandy-like odor	0.023	0.590	0.04	3.889E-02
nonyl acetate		97.1	22.5408	143-13-5	Floral, fruity odor	0.021	0.008	2.63	2.589E-11
7,7-dimethyl-2-bicyclo[3.1.1]heptanyl)methyl acetate	myrtanyl acetate	80	24.5669	29021-36-1		0.017	0.017	1.00	3.528E-01
dodecyl acetate		70.1	29.8252	70808-58-1	Waxy, citrus-rose odor	0.015	0.002	7.50	2.659E-09
methyl (E)-3-phenylprop-2-enoate [Methyl (E)-cinnamate]		161	22.5682	1754-62-7	Fruity balsamic odor	0.015	0.003	5.00	8.742E-07
4-(2,6,6-trimethylcyclohex-2-en-1-yl)butan-2-yl acetate		136.1	29.0433	not available		0.014	0.001	14.00	4.276E-10
1-O-cyclobutyl 2-O-(2-methylpropyl) benzene-1,2-dicarboxylate		149	35.347	not available		0.013	0.025	0.52	8.367E-02
nonan-2-yl butanoate		126.1	25.0083	not available		0.001	0.032	0.03	3.438E-04
Sub total						45.921	25.243	1.82	

**Table 3:** **Terpene molecules identified in the headspace of PdT and RdV berries by GC-MS**.

**Terpene compound**		**Quantification Ion**	**Retention time**	**CAS**	**Descriptor**	**Relative Content (%)**		**fold change**	***p*-value**
**IUPAC name**	**Synonym**					**PdT**	**RdV**		
7,7-dimethyl-4-bicyclo[3.1.1]hept-3-enyl)methyl acetate	myrtenyl acetate	91.1	23.0091	36203-31-3	Pleasant, refreshing, sweet-herbaceous	20.819	15.394	1.35	2.068E-03
7,7-dimethylbicyclo[3.1.1]hept-3-ene-4-carbaldehyde	myrtenal	107	19.4862	57526-63-3	Refreshing, spicy-herbaceous odor	0.765	0.503	1.52	4.039E-06
4-prop-1-en-2-ylcyclohexen-1-yl)methyl acetate	perillyl acetate	91.1	25.7741	5111-96-3	Warm-herbaceous spicy odor	0.464	0.246	1.89	1.492E-07
4-methyl-1-propan-2-ylcyclohex-3-en-1-ol	4-carvomenthenol	71.1	18.9548	562-74-3	Warm-peppery, mildly earthy, musty-woody odor	0.357	0.507	0.70	1.156E-03
(5-methylidene-6-prop-1-en-2-ylcyclohex-3-en-1-yl) acetate		117	32.0095	not available		0.223	0.003	74.33	2.140E-10
1-methyl-2-propan-2-ylbenzene	o-cymene	119.1	14.3651	95660-61-0	Citrusy aroma reminiscent of lemon	0.166	0.502	0.33	1.280E-04
(4-methyl-1-propan-2-yl-4-bicyclo[3.1.0]hexanyl) acetate		59.1	19.3391	not available		0.150	0.492	0.30	2.085E-05
4,7,7-trimethylbicyclo[3.1.1]hept-3-ene	α-pinene	94.1	11.4057	80-56-8	Warm, resinous, pine-like aroma	0.114	0.534	0.21	6.476E-03
1,1,3a-trimethyl-7-methylidene-1a,2,3,4,5,6,7a,7b-octahydrocyclopropa[a]naphthalene	1H-Cyclopropa[a]naphthalene	204.2	28.3446	not available		0.108	0.001	108.00	3.190E-03
1-methyl-4-prop-1-en-2-ylcyclohexene	limonene	93.1	14.4629	95327-98-3	fresh, light, sweet, citrusy aroma	0.094	0.180	0.52	2.519E-04
3,7-dimethylocta-1,6-dien-3-ol	linalool	71.1	16.5999	8008-26-2	pleasant, floral odor	0.063	1.182	0.05	1.523E-06
4-methylidene-1-propan-2-ylbicyclo[3.1.0]hexane	sabinene	93.1	12.7934	3387-41-5	terpineol odor	0.042	0.135	0.31	6.104E-03
4-methyl-1-propan-2-ylbicyclo[3.1.0]hex-3-ene	α-thujene	92	11.4178	75715-79-6		0.041	0.194	0.21	5.281E-03
4E,6E)-2,6-dimethylocta-2,4,6-trien	alloocimene	121.1	16.3138	673-84-7	Warm herbaceous aroma	0.030	0.149	0.20	1.899E-04
3,7,7-trimethylbicyclo[4.1.0]hept-4-ene	4-carene	136.1	16.3028	not available	fruity aroma	0.023	0.090	0.26	1.892E-04
(1S,2S,5R)-2,7,7-trimethylbicyclo[3.1.1]heptan-3-one	isopinocamphone	95.1	18.8561	15358-88-0	cedar camphor aroma	0.023	0.028	0.82	3.825E-02
4-[(1E)-buta-1,3-dienyl]-3,5,5-trimethylcyclohexene	megastigma-3,7(E),9-triene	105	20.0655	not available		0.018	0.001	18.00	6.869E-06
7,7-dimethyl-4-methylidenebicyclo[3.1.1]heptan-3-ol	pinocarveol	92.1	17.8485	5947-36-4	Warm, woody-balsamic aroma	0.016	0.090	0.18	2.921E-09
1-methyl-4-propan-2-ylcyclohexa-1,4-diene	γ-terpinene	93.1	14.4665	99-85-4	Refreshing, herbaceous-citrusy aroma	0.015	0.085	0.18	2.741E-09
3,7-dimethyloct-6-en-1-ol	α-citronellol	69.1	20.313	106-22-9	rose-like aroma	0.006	0.481	0.01	1.305E-08
4-methyl-2,3,4,5,6,7-hexahydro-1H-indene		136.1	23.228	not available		0.049	0.097	0.51	1.557E-06
4,7-dimethyl-1-propan-2-yl-1,2,4a,5,6,8a-hexahydronaphthalene	zizanene	204.1	27.4861	483-75-0		0.012	0.004	3.00	1.294E-03
(1Z,4Z,7Z)-1,5,9,9-tetramethylcycloundeca-1,4,7-triene		80.1	26.4092	not available		0.002	0.016	0.13	9.112E-08
1-ethenyl-1-methyl-2,4-di(prop-1-en-2-yl)cyclohexane		176.1	24.8102	515-13-9		0.000	0.001	0.02	4.165E-04
Sub total						23.601	20.915	1.13	

**Table 4:** **Alcohol and aldehyde molecules identified in the headspace of PdT and RdV berries by GC-MS**.

**Compound**		**Quantification Ion**	**Retention time**	**CAS**	**Descriptor**	**Relative Content (%)**		**fold change**	***p*-value**
**IUPAC name**	**Synonym**					**PdT**	**RdV**		
**ALCOHOLS**									
hexan-1-ol	1-hexanol	56.1	9.3234	25917-35-5	Ethereal, fruity, alcoholic, sweet green aroma	4.990	1.386	3.60	1.084E-04
cyclohexanol		82.1	9.2826	108-93-0	Menthol-like camphorous odor	0.696	0.272	2.56	9.449E-04
octan-1-ol		56.1	15.7268	72-69-5	Sharp fatty-citrus odor	0.661	0.242	2.73	2.226E-04
tridecan-2-ol		45.1	27.3706	67989-40-6		0.163	0.035	4.66	1.890E-05
decan-1-ol		55.1	21.523	85566-12-7	Floral, waxy, fruity odor	0.008	0.084	0.10	5.441E-05
Sub-total						6.518	2.019	3.23	
**ALDEHYDES**									
(E)-hex-2-enal	2-hexenal	98.1	8.7726	73543-95-0	Strong fruity, green, vegetable-like aroma	2.672	5.426	0.49	1.123E-06
(2E,4E)-hexa-2,4-diena	2,4-hexadienal	81	10.7482	80466-34-8	Sweet-green aroma	0.741	1.681	0.44	5.828E-08
hexanal		57.1	7.5617	9012-63-9	Fatty-green, grassy odor	0.699	1.039	0.67	1.826E-03
(E)-oct-2-enal	trans-2-octen-1-al	83	15.3431	2548-87-0	Fatty, green aroma	0.676	0.042	16.10	1.114E-06
nonanal		57.1	16.7129	75718-12-6	Fruity odor	0.673	0.444	1.52	4.207E-03
2,2-dimethylhexanal		57	12.9171	not available		0.149	0.178	0.84	1.226E-01
2-phenylacetaldehyde	benzeneacetaldehyde	91	14.984	122-78-1	Very powerful and penetrating pungent green floral and sweet odor of hyacinth type	0.069	0.062	1.11	2.931E-01
(E)-non-2-ena	2-nonenal, (E)-	43.1	18.4043	30551-15-6	Powerful, penetrating fatty, violet aroma	0.039	0.191	0.20	4.341E-03
decanal		70.1	19.7005	75718-12-6	Fatty, floral-orange odor	0.023	0.021	1.10	2.257E-01
Sub total						5.741	9.084	0.63	

**Table 5:** **Ketone and lactone molecules identified in the headspace of PdT and RdV berries by GC-MS**.

**Compoun/d**		**Quantification Ion**	**Retention time**	**CAS**	**Descriptor**	**Relative Content (%)**		**fold change**	***p*-value**
**IUPAC name**	**Synonym**					**PdT**	**RdV**		
**KETONES**									
tridecan-2-one	2-tridecanone	58.1	27.1904	593-08-8	Milky, herbaceous, slightly spicy odor	2.277	0.644	3.54	4.828E-09
heptan-2-one	2-heptanone	58.1	9.9628	29308-56-3	Fruity, spicy odor	0.662	12.694	0.05	7.379E-07
undecan-2-one	2-undecanone	58.1	22.0932	112-12-9	Citrus, fatty, rue-like odor	0.510	1.182	0.43	4.140E-04
nonan-2-one	2-nonanone	43.1	16.3408	821-55-6	Fruity, floral, fatty, herbaceous odor	0.240	11.953	0.02	8.818E-08
pentadecan-2-one	2-pentadecanone	71.1	31.7836	2345-28-0		0.036	0.043	0.84	1.232E-01
1-phenylethanone	acetophenone	105	15.7516	98-86-2		0.031	0.099	0.31	7.649E-05
Sub total						3.756	26.615	0.14	
**LACTONES AND FURANONES**									
4-methoxy-2,5-dimethylfuran-3-one	mesifurane (DMF)	142	15.3621	4077-47-8	Sweet, carmellic, burnt sugar, aroma	0.869	0.001	869.00	1.145E-06
5-hexyloxolan-2-one	γ-decalactone	85	26.5909	706-14-9	Fruity, peach-like odor	0.391	0.376	1.04	4.634E-01
5-pentyloxolan-2-one	γ-nonalactone	85	21.22	82373-92-0	Coconut-like odor	0.155	0.032	4.84	1.750E-07
5-ethyloxolan-2-one	γ-hexalactone	85	15.231	695-06-7	Herbaceous, sweet odor	0.030	0.022	1.36	1.957E-01
Sub total						1.445	0.431	3.35	

The relative abundance of individual chemical classes significantly differed between the two strawberries (Supplementary Figure 1). Quantitatively, esters were the predominant volatiles in berries of PdT, accounting for nearly 50% of the aroma. In comparison, their relative amount was drastically reduced in the aroma of RdV, only representing 25% of the total volatiles. Terpenes were the second most abundant class of compounds found in PdT (24%). A comparable level of total terpenes was identified in RdV (21%). Conversely, the two strawberries disclosed a marked difference in the relative level of ketones, which were severely reduced in PdT (4%), while highly abundant in RdV (27%). We also observed a disparity in the relative abundance of alcohols, which were far more copious in PdT compared with RdV, representing 7and 2% of the total volatile molecules, respectively. No substantial differences were detected in the relative amount of aldehydes and lactones. Aldehydes accounted for 6 and 9% of total volatiles in PdT and RdV, respectively, whilst lactones were the least represented molecules, only covering 1.4 and 0.4% of the aroma of PdT and RdV, respectively. Finally, compounds of uncertain identity were slightly more abundant in RdV (14%) compared with PdT (8%) (Supplementary Figure 1).

Analysis of individual constituents of the aroma identified the monoterpene myrtenyl acetate as the most abundant volatile in both PdT and RdV (Table 3). This finding is in line with a previous work, which demonstrated that this molecule dominates the terpenoid profile of wild strawberries (Aharoni et al., 2004). Interestingly, the level of myrtenyl acetate was significantly augmented in PdT (20.1%) compared with RdV (15.4%). The relative quantities of two esters molecules were also preeminent in the aroma of PdT, namely octyl acetate (12.7%) and 4-acetyloxybutyl acetate (11.6%) (Table 2). Together with myrtenyl acetate these two compounds constituted 45% of the total volatiles found in this strawberry. Both esters were also detected in the aroma of RdV, even tough their relative abundance was drastically reduced compared with PdT (4.6 and 3.6%, respectively). Two ketones, 2-heptanone, and 2-nonanone, were the most abundant molecules, after myrtenyl acetate, in the aroma of RdV, accounting for 12.7 and 11.9% of the total volatiles, respectively (Table 5). Conversely, the amount of these two molecules was significantly reduced in PdT (0.7 and 0.2%, respectively).

Additional abundant components of the aroma of PdT included, 1-hexanol (5%) (Table 4) and several other esters, as hexyl formate (4.5%), methyl anthranilate (MA) (3.4%) and hexyl acetate (2.7%) (Table 2). On average, the content of these volatiles was significantly higher in the aroma of PdT compared with RdV. The only aldehyde found in relatively high amounts in both strawberries was 2-hexenal (Table 4). Its content was significantly greater in RdV (5.4%) compared with PdT (2.7%). The most abundant ketone in the aroma of PdT was 2-tridecanone, reaching 2.3% of the total volatiles (Table 5). As opposite to the other ketones, the level of this molecule was significantly reduced in RdV (0.6%) compared with PdT.

Among less abundant molecules (<1% of total volatiles), major differences between the two strawberries were observed within terpenes, esters, and furanones. In particular, the levels of α-pinene, a monterpene specifically identified in the aroma of *F. vesca* (Aharoni et al., 2004), linalool, known to dominate the terpenoid profile of cultivated strawberry (Aharoni et al., 2004) and α-citronellol, were significantly reduced in the aroma of PdT in comparison with RdV (Table 3). Contrariwise, the level of megastigma-3,7(E),9-triene, the major terpene found in the essential oil of some Eucalyptus species (El-Mageed et al., 2011), was 18 times higher in PdT compared with RdV. We also observed a 74- and 108-fold increase in the accumulation of the terpenes 3-cyclohexen-1-ol,5-methylene-6-(1-methylethyl)-(9CI) and 1H-Cyclopropa[a]naphthalene, in the aroma of RdV compared with PdT (Table 3). Among minor esters, the most striking differences were detected for 2-methylbutanoic acid and methyl 2-methylbutanoate, whose content was 120- and 150-times more abundant in berries from PdT relatively to RdV, respectively (Table 2). Additional esters over-represented in the aroma of RdV included [(E)-3-phenylprop-2-enyl] acetate (24-fold), octyl 3-methylbutanoate (16-fold), tridecan-2-yl acetate (14-fold) and methyl tiglate (13-fold) (Table 2).

Even if present in lower amount compared to other volatiles, lactones and furanones, were more copious in the aroma of PdT compared to RdV. Remarkably, mesifurane, the typical furanone conferring sweet caramel notes to wild strawberries, was nearly 900 times more abundant in PdT compared with RdV (Table 5).

### Analysis of SAAT expression in developing berries

In strawberries only very few genes have been directly associated with aroma biogenesis in ripening fruits. Among them, the *Strawberry Alcohol Acyltransferase* gene (*SAAT*), controlling a key step in esters biosynthesis (Aharoni et al., 2000), is of particular interest. The enzyme AAT catalyzes the transfer of an acyl moiety from acyl-CoA onto specific alcohols, resulting in the production of ester molecules (Harada et al., 1985). Intriguingly, octyl-acetate, the most abundant ester in both PdT and RdV aroma, has been demonstrated to be a genuine AAT product (Aharoni et al., 2000). The activation of *SAAT* expression during berry development has been positively correlated with the on-set of esters accumulation. In *F*. × ananassa *SAAT* expression is induced at early stage during fruit ripening, it peaks in correspondence of the turning stage, and it is rapidly down-regulated in red fruits (Aharoni et al., 2000). We compared the expression profile of the *SAAT* gene in developing berries from PdT and RdV (Supplementary Figure 2) to unravel potential differences in the level of gene expression and/or in the kinetic of *SAAT* activation between the two strawberries.

**Figure 2:**
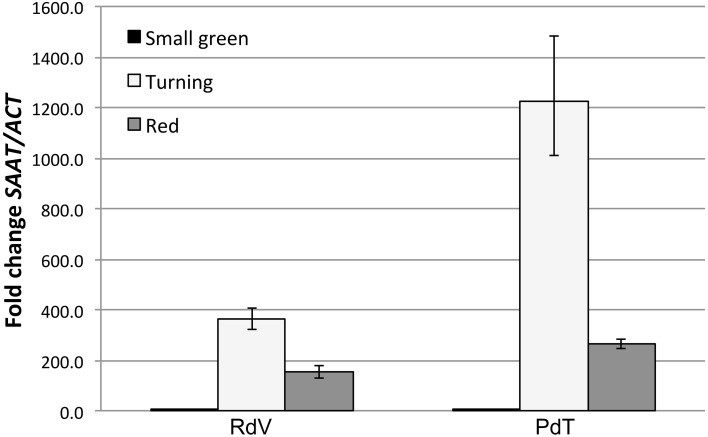
**Comparative analysis of *SAAT* gene expression in developing berries**. qPCR analysis of *SAAT* expression in small green, turning and red fruits of RdV and PdT. Relative *SAAT* transcript levels were determined using *SAAT*-specific primers and normalized to the expression of a strawberry *ACTIN* gene (GenBank: JN616288.1).

As shown in Figure 2, both RdV and PdT accumulated comparable low levels of *SAAT* transcripts in small green fruits. At the turning stage, both strawberries displayed very strong activation of *SAAT* expression. Interestingly, at this stage, the degree of gene activation was significantly enhanced in PdT compared with RdV (*t*-test, *p* < 0.01). This finding is conceivable with the increased accumulation of esters observed in PdT relatively to RdV fruits. Finally, in red fruits *SAAT* expression was down-regulated to the same extent in both berries (Figure 2).

## Discussion

The vast array of wild species found in the genus *Fragaria* provide an exceptionally large and conveniently located germplasm, which can serve to breeding novel quality traits into garden strawberries (Hancock and Luby, 1993). Key to the successful exploitation of the natural variation occurring in the *Fragaria* genus is the detailed characterization of the aroma profile of individual species, ecotypes and clones. This study unraveled the volatile composition of two domesticated wild strawberries: *F. moschata* clone Profumata di Tortona and *F. vesca* cv. Regina delle Valli. Both strawberries are regarded as highly aromatic, although the scent of PdT is far more intense and persistent (Urruty et al., 2002).

Sampling procedures are key to the analysis of aroma composition in strawberries, as significant changes in VOCs profiles can occur among harvest dates within a single season (Schwieterman et al., 2014). Different strategies employing multiple harvests or the laborious harvest of all the available fruits throughout the season have been proposed to overcome this obstacle (Ulrich and Olbricht, 2013; Schwieterman et al., 2014). Our results rely on the analysis of biological replicas from a single harvest. In contrast to perpetual flowering accession characterized by prolonged harvesting seasons, the seasonal flowering PdT clone, only bears fruit for less than 2 weeks. Environmental changes and differences in plant physiology, known to affect fruit quality over months (Schwieterman et al., 2014), are unlike to influence the aroma composition of PdT berries within a period of 15 days.

We identified 131 volatiles in ripe berries of PdT and RdV, a number exceeding the aroma compounds usually found in commercial strawberries. A recent survey of the chemical diversity of the aromas of 35 different garden varieties recognized no more than 80 VOCs even in the most fragrant commercial genotypes (Schwieterman et al., 2014). Comparative analysis of aroma patterns revealed the significant enrichment in esters and alcohols along with the severe reduction in ketones in berries from PdT compared with RdV. Conversely, the two strawberries disclosed comparable levels of terpenes, aldehydes and lactones (Supplementary Figure 1). Over 130 different ester molecules have been identified in strawberries (Latrasse, 1991). In *F. ×* ananassa, the chemical composition of ester volatiles is usually dominated by methyl and ethyl esters even though the abundance of each form varies with cultivar (Forney et al., 2000). Methyl esters were the prevalent form in the aroma of both PdT and RdV (22 molecules), whereas ethyl esters were poorly represented (3 molecules) (Table 2).

Even if it is generally difficult to establish a direct correlation between individual aroma constituents and specific sensory impressions, the fragrance of wild strawberries largely depend upon the relatively high amounts of methyl anthranilate (MA) (Ulrich et al., 1997). The intensive sweetish-flowery impression of this ester is at the basis of the definition of strawberry aroma chemo-types, which are essentially subdivided into MA-containing and MA-free types (Ulrich et al., 1997). Our analysis uncovered a nine-fold increase in the level of MA in berries from PdT compared with RdV, thus emphasizing the role of this key ester in determining the unique fragrance of musk strawberry. This conclusion is corroborated by previous studies, which reported an exceedingly higher concentration of MA in *F. moschata* relatively to *F. vesca* (Urruty et al., 2002). It is also interesting to note that, MA and γ-decalactone can directly inhibit the *in vitro* growth of relevant strawberry pathogens, thus implying that these volatiles may contribute to the healthiness of the berry at harvest (Chambers et al., 2013).

We also detected significant differences in the accumulation of low abundant esters between the two strawberries, as for instance methyl 2-methylbutanoate, which was 150 times more abundant in PdT compared with RdV (Table 2). Along with other esters of butanoic acid this molecule, conferring a sweet and fruity impact to the aroma, is found in higher amounts in garden strawberries compared with woodland accessions (Ulrich and Olbricht, 2013). We also detected a 16-fold increase in the relative level of octyl 3-methylbutanoate, in the headspace of PdT compared with RdV. This molecule, conferring an apple-pineapple odor, is present in the most flavored commercial varieties but undetected in the least flavorful. Evidence indicates that, octyl 3-methylbutanoate is an important component of strawberry aroma, potentially enhancing the perceived sweetness intensity, independently of individual sugars (Schwieterman et al., 2014).

Ester accumulation in ripening strawberries is directly associated with the expression of the *SAAT* gene, encoding a fruit-specific ALCOHOL ACYLTRANSFERASE (AAT) (Aharoni et al., 2000). It is intriguingly to speculate that the enhanced accumulation of ester molecules in the aroma of PdT, results from the hyper activation of *SAAT* expression observed in turning fruits from PdT, as compared with RdV (Figure 2). Further support to this hypothesis comes from the observation that genuine products of the SAAT enzyme, including octyl acetate, [(Z)-hex-3-enyl] acetate, 2-phenylethyl acetate and nonyl acetate (Aharoni et al., 2000), were found at higher levels in the headspace of PdT compared with RdV (Table 2).

The implication for the increased alcohol accumulation in PdT, as compared with RdV, on the quality of the berry is questionable, as these molecules have normally little impact on the aroma (Larsen and Watkins, 1995). Nevertheless, Schwieterman and colleagues recently reported a direct effect of the level of 1-hexanol on sweetness and flavor intensity in different garden cultivars (Schwieterman et al., 2014). Notably, we found a significant 4-fold increase in the relative amount of 1-hexanol in PdT compared with RdV, possibly suggesting a positive role for this molecule in determining the unique flavor of musk strawberries.

PdT and RdV displayed a similar terpene profile, largely dominated by myrtenyl acetate, by far the most abundant molecule found in the aroma of both strawberries. Higher concentrations of myrtenyl acetate are normally found in woodland strawberries compared with garden varieties (Ulrich and Olbricht, 2013). We observed a moderate, although significant, increased level of myrtenyl acetate in PdT compared with RdV (Table 3). This is in accord with previous comparative analysis of other *F. moschata* clones with *F. vesca* accessions (Ulrich et al., 1997). Major differences in the accumulation of low abundant terpenes involved linalool and 1H-Cyclopropa[a]naphthalene. The former, conferring pleasant flowery, citrus-like notes, represents the predominant monoterpene found in cultivated strawberries (Aharoni et al., 2004; Ulrich and Olbricht, 2014). Its relative concentration was 19 fold higher in RdV compared with PdT (Table 3). Conversely, 1H-Cyclopropa[a]naphthalene was 108 times more abundant in PdT in comparison with RdV. Interestingly, this molecule is among the major constituent of some agarwood oils, highly appreciated for their unique and intense fragrance and for their therapeutic properties (Takemoto et al., 2008). To our knowledge, this volatile compound has never been reported in previous analyses of strawberry aromas and could represent a novel target to enhancing the fragrance of traded strawberries.

Even if present in relative low amounts, furanones are considered as dominating components of strawberry aroma. In particular furaneol (DHF) and its methyl ether mesifurane (DMF), contribute to the typical caramel-like, sweet, floral and fruity aroma of the berry (Jetti et al., 2007). Interestingly, PdT strawberries revealed an enhanced accumulation of total furanones compared with RdT. In particular, we detected a nearly 900-fold increase in the relative amount of DMF compared with RdV (Table 5). Augmented levels of DMF in *F. moschata* accessions relatively to *F. vesca* have been previously reported, yet not to this very large extent (Ulrich et al., 1997). The identification of the genetic bases for such an increased mesifurane production is beyond the scope of this work. Yet it is important to note that, PdT may represent a valuable breeding material to enhance the DMF content of garden varieties. Our analysis did not reveal detectable amount of DHF neither in PdT nor in RdV berries. This is in contrast with a preceding work, which identified this furanone in different *F. vesca* and *F. moschata* genotypes (Urruty et al., 2002). One possible explanation for such a discrepancy could reside in differences in the analytic methods employed in previous studies and ours. It is interesting to note, that DHF accumulation has been negatively correlated to the quality of the berry, as DHF-type strawberries are generally characterized by medium to poor flavor (Ulrich et al., 1997). The lack of DHF in PdT and RdV berries could alternatively be correlated to the organoleptic excellence of their fruits.

As a whole, our data provide a comprehensive metabolic map of PdT, the most fragrant strawberry of all. Despite the fact that *F. moschata* is not a direct ancestor of commercial garden strawberries, the aroma profile of PdT could assist the exploitation of this ancient clone as breeding material to enhance fruit quality in commercial strawberries. The successful introgression of wild-derived traits into cultivated garden varieties largely depends upon the possibility to generate viable and fertile interspecific hybrids. Evidently, crosses between octoploid *F*. × ananassa and species at lower ploidy level, including hexaploid PdT, are rather difficult. Yet, breeders have successfully performed interploid crosses between cultivated strawberries with *F. vesca* and *F. moschata*, which produced viable hybrids with partial seed set (Luby et al., 1991). Synthetic octoploids containing varying levels of *F. moschata* have also been generated using colchicine-induced artificial doubling of chromosome number (Evans, 1982a,b). Finally, advancements in genetic engineering of cultivated strawberries have opened unprecedented possibilities for the breeding of new varieties with desirable traits. Genetic transformation has been successfully employed to enhance strawberry resistance to pests, herbicides, diseases, environmental stresses as well as fruit quality (reviewed in Qin et al., 2008). Further developments are expected in which metabolomic data, as those provided in this study, combined with genome-wide transcriptomic analysis and next generation genome-sequencing strategies will allow the identification of suitable molecular targets for engineering of volatile biosynthesis in strawberries. In this perspective, the genome of Profumata di Tortona will prove an invaluable source of genetic material.

### Conflict of interest statement

The authors declare that the research was conducted in the absence of any commercial or financial relationships that could be construed as a potential conflict of interest.
