# Supplementary material for: Comparative analysis of fruit aroma patterns in the domesticated wild strawberries “Profumata di Tortona” (F. moschata) and “Regina delle Valli” (F. vesca)
Source: Front Plant Sci. 2015 Feb 11;6:56. doi: 10.3389/fpls.2015.00056 (PMC4324068; doi:10.3389/fpls.2015.00056)
Supplement: Supplementary file 1 [file Image1.PDF]

Supplementary Material

Comparative analysis of fruit aroma patterns in the domesticated wild strawberries "Profumata di Tortona" (*F. moschata*) and "Regina delle Valli" (*F. vesca*).

Alfredo S. Negri[1], Domenico Allegra[2], Laura Simoni[2], Fabio Rusconi [2], Chiara Tonelli [3], Luca Espen [1]*, Massimo Galbiati [2,3]*

[1]Dipartimento di Scienze Agrarie e Ambientali - Produzione, Territorio, Agroenergia, Università degli Studi di Milano, Milano, Italy
[2]Fondazione Filarete, Milano, Italy
[3]Department of Life Sciences, Università degli Studi di Milano, Milano, Italy

***Correspondence:** Massimo Galbiati, Department of Life Sciences, Università degli Studi di Milano, Via Celoria 26, Milano, 20133, Italy.
massimo.galbiati@unimi.it
Luca Espen, Dipartimento di Scienze Agrarie e Ambientali - Produzione, Territorio, Agroenergia, Università degli Studi di Milano, Via Celoria 2, Milano, 20133, Italy.
luca.espen@unimi.it

1. Supplementary Figures and Tables

1.1. Supplementary Figure 1

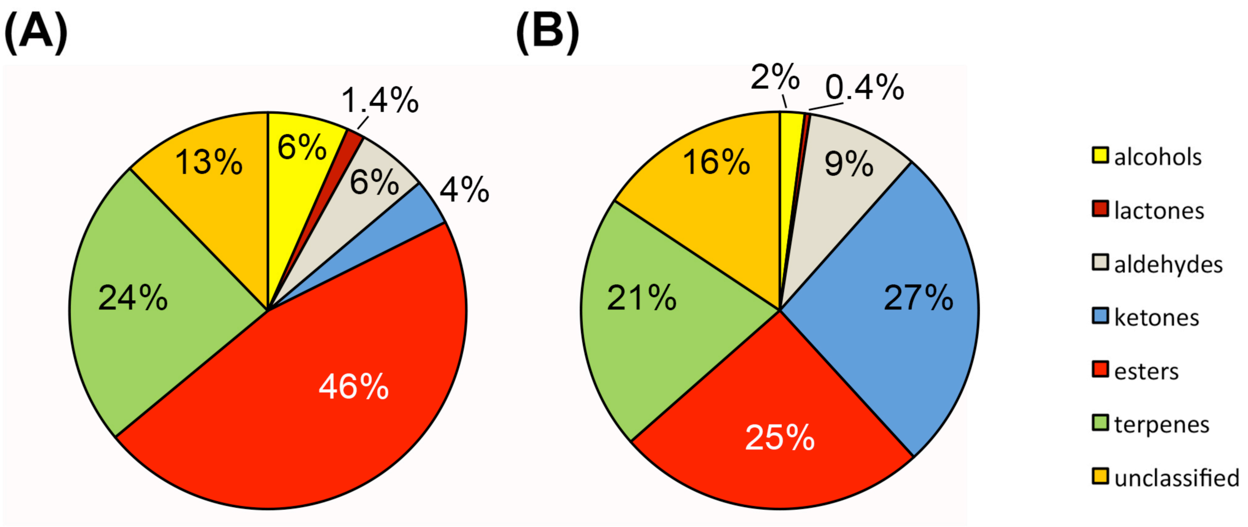

Supplementary Figure 1. Relative abundance of volatiles grouped by chemical classes. (A) Volatile composition of *F. moschata*, clone Profumata di Tortona. **(B)** Volatile composition of *F. vesca*, cv Regina delle Valli. Percentages represent the sum of the relative abundance of single molecules identified in each chemical class.

1.2. Supplementary Figure 2

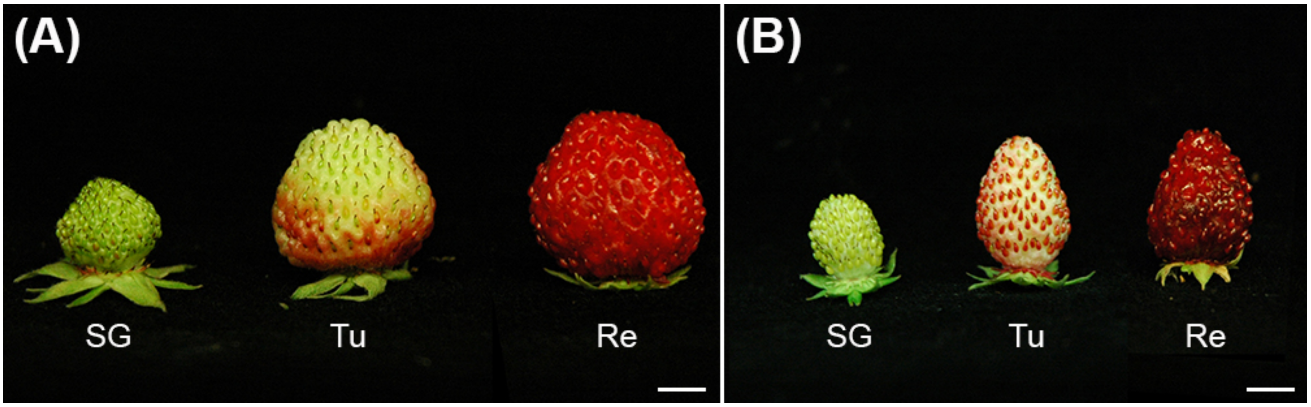

Supplementary Figure 2. Strawberry fruits development and ripening. **(A)** *F. moschata*, clone Profumata di Tortona. **(B)** *F. vesca*, cv Regina delle Valli. Developmental stages were identified as indicated by Fait *et al*. (2008). SG, small green; Tu, turning; Re, red. Scale bar = 1 mm.

2. References[1]

Fait, A., Hanhineva, K., Beleggia, R., Dai, N., Rogachev, I., Nikiforova, V.J., Fernie, A.R., and Aharoni, A. (2008). Reconfiguration of the achene and receptacle metabolic networks during strawberry fruit development. *Plant Physiol* 148**,** 730-750. doi: 10.1104/pp.108.120691.

[1] Provide the doi when available, and ALL complete author names.
